# Develop an efficient and specific AAV-based labeling system for Muller glia in mice

**DOI:** 10.1038/s41598-022-27013-0

**Published:** 2022-12-27

**Authors:** Yanxia Gao, Kailun Fang, Zixiang Yan, Haiwei Zhang, Guannan Geng, Weiwei Wu, Ding Xu, Heng Zhang, Na Zhong, Qifang Wang, Minqing Cai, Erwei Zuo, Hui Yang

**Affiliations:** 1 Institute of Neuroscience, CAS Center for Excellence in Brain Science and Intelligence Technology, Shanghai Research Center for Brain Science and Brain-Inspired Intelligence, Chinese Academy of Sciences, 320 Yue Yang Road, Shanghai, 200031 People’s Republic of China; 2 Shenzhen Branch, Guangdong Laboratory for Lingnan Modern Agriculture, Genome Analysis Laboratory of the Ministry of Agriculture, Agricultural Genomics Institute at Shenzhen, Chinese Academy of Agricultural Sciences, Shenzhen, China; 3 Huigene Therapeutics Inc., Shanghai, China; 4 Department of Vascular Surgery, Changhai Hospital, Navy Medical University, Shanghai, People’s Republic of China; 5 Department of Vascular Surgery, The Affiliated Hospital of Qingdao University, Qingdao, China

**Keywords:** Differentiation, Neurogenesis, Retina

## Abstract

Reprogramming Müller glia (MG) into functional cells is considered a promising therapeutic strategy to treat ocular diseases and vision loss. However, current AAV-based system for MG-tracing was reported to have high leakage in recent studies. Here, we focused on reducing the leakage of AAV-based labeling systems and found that different AAV serotypes showed a range of efficiency and specificity in labeling MG, leading us to optimize *a human GFAP-Cre* reporter system packaged in the AAV9 serotype with the *woodchuck hepatitis virus post-transcriptional regulatory element* (WPRE) removed. The leakage ratio of the AAV9*-hGFAP-Cre-*Δ*WPRE* decreased by an approximate 40-fold compared with the AAV9*-hGFAP-Cre-WPRE* labeling system. In addition, we validated the specificity of the AAV-ΔWPRE system for tracing MG reprogramming under Ptbp1-suppression and observed strict non-MG-conversion, similar to previous studies using genetic lineage tracking mouse models. Thus, the *AAV9-hGFAP-Cre-*Δ*WPRE* system showed high efficiency and specificity for MG labeling, providing a promising tool for tracing cell fate in vivo.

## Introduction

Muller glia (MG) represents the main type of glial cell which are responsible for maintaining retinal structure and support for neurons in the retina. In lower vertebrates (e.g. zebrafish, *Danio rerio*), MG can re-enter the cell cycle to proliferate and subsequently differentiate into multiple cell types following injury, such as photoreceptors or retinal ganglion cells (RGCs)^1–4^, showing great potential for the treatment of retinal degenerative diseases. However, this process does not occur in mammals. Decades of research efforts have been committed to investigating the regenerative machinery in adult mammals with the ultimate goal of inducing MG regeneration and reprogramming. Several major insights have emerged from this work. For example, Ascl1 overexpression or simultaneous deletion of three nuclear factors I (NFI) genes can both induce MG proliferation and transformation into amacrines or bipolar cells in mice^5,6^. Similarly, MG conversion to RGCs was also observed in mice following the deletion of Ptbp1 or simultaneous ectopic expression of Pou4f2 (Brn3b) and Atoh7 (Math5)^7,8^. Moreover, photoreceptors could be generated from MG under simultaneous overexpression of Otx2, Crx, and Nrl after a period of β-catenin ectopic expression^9^.

However, whether MG was converted into functional neurons remains controversial due to the leakage of the MG labeling system. For example, in Ptbp1-knockdown groups, significantly more reporter^+^ neurons were observed by the AAV-pGFAP-reporter tracing system, while this MG conversion was verified as an artifact when the genetic lineage tracking system (e.g. Glast-CreERT; Rosa-CAG-LSL-Sun1-GFP) was used to label MG^10,11^.

Although genetic lineage tracing systems are more stringent than AAV-pGFAP-reporter^10–14^, AAV-based systems still have potential in application. For example, it is convenient for AAV-based systems to be performed without crossing mouse strains, and it can be applied to various animal species having no genetic strains, such as non-human primates. Considering the major defect of AAV-based system is the high level of leakage^10–12^, we thus focused on optimizing AAV-based system to reduce the leakage.

Here, to increase the specificity of the AAV-based MG-tracing system, we screened different AAV serotypes for MG transduction, and eventually validated AAV9 as the best serotype for MG labeling with high transduction efficacy and low leakage. In addition, we removed the WPRE (woodchuck hepatitis virus post-transcriptional regulatory element) element in current MG label systems. Combine AAV9 and the Δ*WPRE* construct, we provided a highly specific, and reproducible labeling system for MG cells in mouse retina. Finally, we found that the results of MG-conversion using AAV9-Δ*WPRE* labeling system was consistent with those using genetic lineage tracing systems reported in previous studies.

## Results

### AAV-based reporter labels MGs efficiently but not specifically

**Figure 1:**
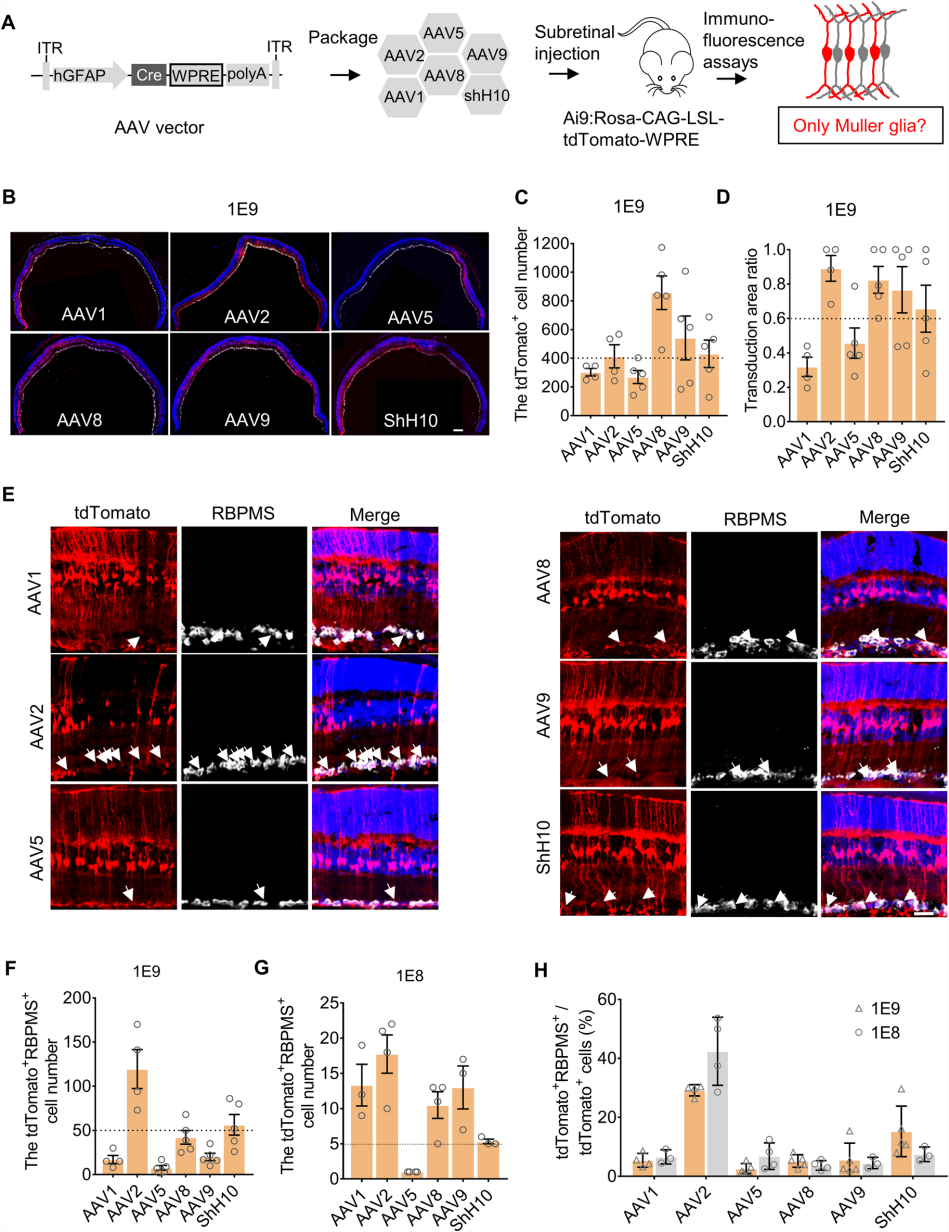
AAV-based systems co-label a small proportion of RGCs along with Muller glia. (**A**) Schematic illustration of experimental design. Cre expression is driven by the human *GFAP* promoter and enhanced by the *WPRE* element. WPRE woodchuck hepatitis virus post-transcriptional regulatory element. (**B**) Representative images of transduction with different AAV serotypes through subretinal injection at the dose of 1 × 10^9^ vector genomes per eye (vg/eye). The whole retina was sectioned and pasted in serial glass slides. Images were chosen from sections near the injection site after staining. Scale bar, 200 μm. (**C**) The average number of tdTomato-labeled cells in each eye section infected by different AAVs at the dose of 1 × 10^9^ vg/eye. (**D**) Quantification of transduction area ratio in each section at the dose of 1 × 10^9^ vg/eye. (**E**) Representative images of co-localization of tdTomato signal with the retina ganglion cell (RGC) marker Rbpms for each AAV serotype at the dose of 1 × 10^9^ vg/eye. Scale Bar, 50 μm. (**F**) The number of tdTomato-labeled RGCs at doses of 1 × 10^9^ vg/eye. (**G**) The number of tdTomato-labeled RGCs at doses of 1 × 10^8^ vg/eye. (**H**) The ratio of tdTomato-labeled RGCs to total labeled cells at doses of 1 × 10^9^ and 1 × 10^8^ vg/eye. n = 3 ~ 5 retinas per group for panels C, D, F, and G from 3 ~ 5 mice. All values are presented as means ± standard error mean (S.E.M.)

AAV-based Cre expression is commonly used to label MG cells in mice due to its relatively easier introduction compared to generating transgenic mouse lines^7–9,15^. However, the transduction efficiency and tropism varied among different AAV serotypes and among different administration routes^16,17^. To evaluate the MG-labeling efficiency of different AAV serotypes driven by the GFAP promoter, we packaged the *hGFAP-Cre-WPRE* construct into commonly used AAV vectors, including AAV1, AAV2, AAV5, AAV8, AAV9 and AAV.ShH10^9,18,19^. Given that low transduction efficiency of these serotypes by intravitreal injection^19^, we delivered them to the retinas of Ai9 mice by subretinal injection (Fig. 1A). We observed distinct transduction patterns among different AAV serotypes at the dose of 1 × 10^9^ vector genomes per eye (vg/eye). In particular, AAV1 and AAV5 showed less diffusion in the retina and their transduction area was smaller than that of other AAVs, while AAV8 and AAV9 transduced more MGs than other AAVs, with > 500 labeled cells per retina section, on average (Fig. 1B–D). Unexpectedly, the tdTomato reporter showed highly variable leakage to RGCs among serotypes (Fig. 1E). Notably, AAV2 and AAV.ShH10 labeled a greater number of RGCs (> 50 cells on average per eye section than other vectors (Fig. 1F).

To decrease the labeling leakage in the RGCs, we used a lower dose of AAVs (1 × 10^8^ vg/eye) to transduce the mouse retina. It is found that the number of tdTomato-labeled RGCs decreased a lot at the dose of 1 × 10^8^ vg/eye (Fig. 1G), but the number of total tdTomato-labeled cells also decreased with the dose (Supplementary Fig. 1A–C). As a result, leakage ratio among total labeled cells was not improved compared to the high dose group (1 × 10^9^ vg/eye) (Fig. 1H). These results implied that the selection of appropriate AAV serotype could improve transduction efficiency and specificity to some extent, but non-specific labeling of RGCs was still existed even in a low viral dosage. Thus, an alternative method was necessary to improve labeling specificity.

### Deletion of the WPRE element in the AAV9 construct eliminates non-specific neuron labeling

**Figure 2:**
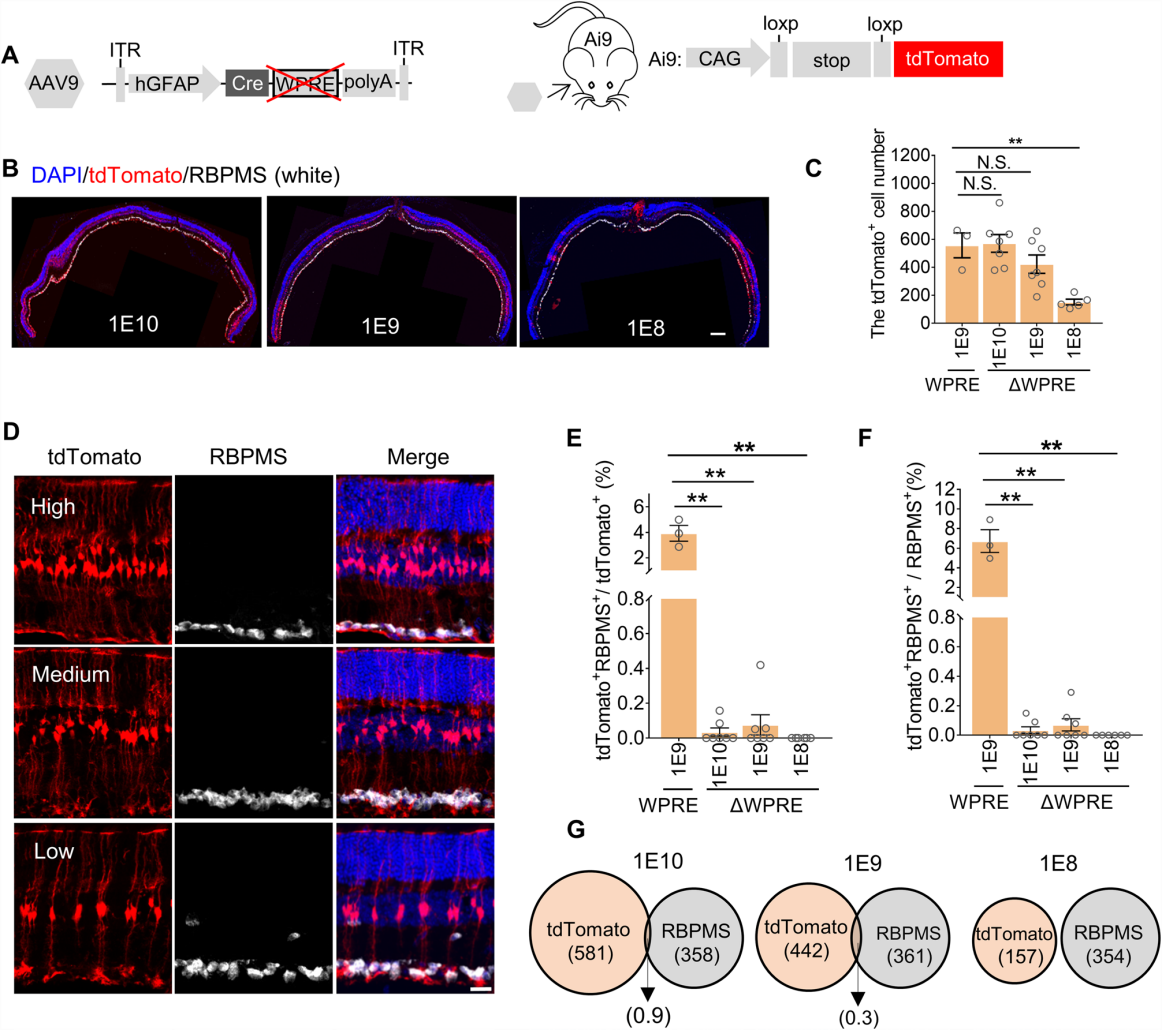
Reduced leakage in the RGCs with the *ΔWPRE* system. (**A**) Schematic diagram of the *hGFAP-Cre-*Δ*WPRE* vector and the tdTomato reporter construct in the Ai9 mouse line. (**B**) Representative images of tdTomato co-localization with Rbpms in mouse retinas following subretinal injection with different doses of AAVs (from left to right:1 × 10^10^, 1 × 10^9^, 1 × 10^8^ vg/eye). Scale bar, 200 μm. (**C**) The average number of tdTomato^+^ cells in each eye section transduced by different doses of AAVs. (**D**) Representative images of tdTomato co-labeling with the RGC marker in different densities of labeled MG in retina sections. Scale Bar, 50 μm. (**E,F**) The ratio of tdTomato-labeled MG among total labeled cells (E) and RGCs (F). 3 eye sections near the injection site for each retina were chosen to count cells, and their average values were shown and analyzed. (**G**) Venn diagram of the average number of tdTomato-labeled cells and RGCs. n = 3 ~ 7 retinas per group for panels C, E, F. All values are presented as means ± S.E.M.

*WPRE* is often used to enhance gene expression in AAV vectors^20–24^, we hypothesized that the enhanced Cre expression due to *WPRE* element could lead to tdTomato activation in cells with low GFAP promoter activity, potentially resulting in non-specific labeling of RGCs by AAV-based reporters targeting MG. To test this hypothesis, we deleted *WPRE* from the reporter construct (Fig. 2A) and packaged it in the AAV9 serotype for delivery by subretinal injection to the eyes of Ai9 mice. We then evaluated the efficiency and MG specificity of tdTomato^+^ labeling. We found that the tdTomato^+^ cell number varied in a dose-dependent manner, with a more extensive reporter signal in the high-dose injection groups (1 × 10^10^ and 1 × 10^9^ vg /eye) and significantly fewer labeled cells in the 1 × 10^8^ group (Fig. 2B,C). However, the tdTomato^+^ cell number was comparable between the *WPRE* and Δ*WPRE* groups at 1 × 10^9^ dose (Fig. 2C). Furthermore, 99%, 98%, and 96% of the total labeled cells separately were co-labeled with the MG marker Sox9 in the 1 × 10^10^, 1 × 10^9^, and 1 × 10^8^ groups, and these tdTomato^+^Sox9^+^ cells respectively accounted for 73.8% ± 8.5%, 51.6% ± 9.4% and 20% ± 4.16% of the total Sox9^+^ cells at these three doses (Supplementary Fig. 2A-C), which indicated that almost all tdTomato^+^ cells are Sox9^+^ MG cells. The deletion of *WPRE* did not significantly influence the efficiency of in vivo MG labeling.

Next, we validated the specificity of Δ*WPRE* system in MG labeling. RBPMs is a marker of RGCs. We observed almost no tdTomato^+^Rbpms^+^ cells in ΔWPRE group (Fig. 2D). Under the dose of 1 × 10^9^ vg/eye, the Δ*WPRE* group showed a 40-fold reduction in the ratio of tdTomato^+^Rbpms^+^ cells to total tdTomato^+^ cells (from 3.93% ± 0.61% to 0.08% ± 0.05%) compared with the WPRE group, and an 80-fold (from 6.72% ± 1.15% to 0.08% ± 0.04%) among total Rbpms^+^ cells (Fig. 2E,F). Less than one RGC (15 tdTomato^+^Rbpms^+^ cells in 22,269 total labeled cells) on average was labeled in each eye section (Fig. 2G). It warrants that the proportion of labeled RGCs remained consistently low across a wide dosage range, 0.08% to 0% from 1 × 10^10^ to 1 × 10^8^ vg per eye in the Δ*WPRE* group. To validate this difference between the *WPRE* and Δ*WPRE* groups is due to the WPRE element but not to the AAV production process, we injected *AAV9-GFAP-Cre-WPRE* and *AAV9-GFAP-Cre* from different AAV facilities and companies and found that leakage in the RGC still existed in the *AAV9-GFAP-Cre-WPRE* comparing with *AAV9-GFAP-Cre*, although tiny differences in transduction efficiency and leaky ratio among batches from different AAV facilities (Supplementary Fig. 2D–G). Collectively, these results indicated that the *ΔWPRE* AAV reporter system could hardly label RGCs, even in experiments requiring high viral titer.

### The ΔWPRE system fails to trace reprogrammed RGCs mediated by transcription factors

**Figure 3:**
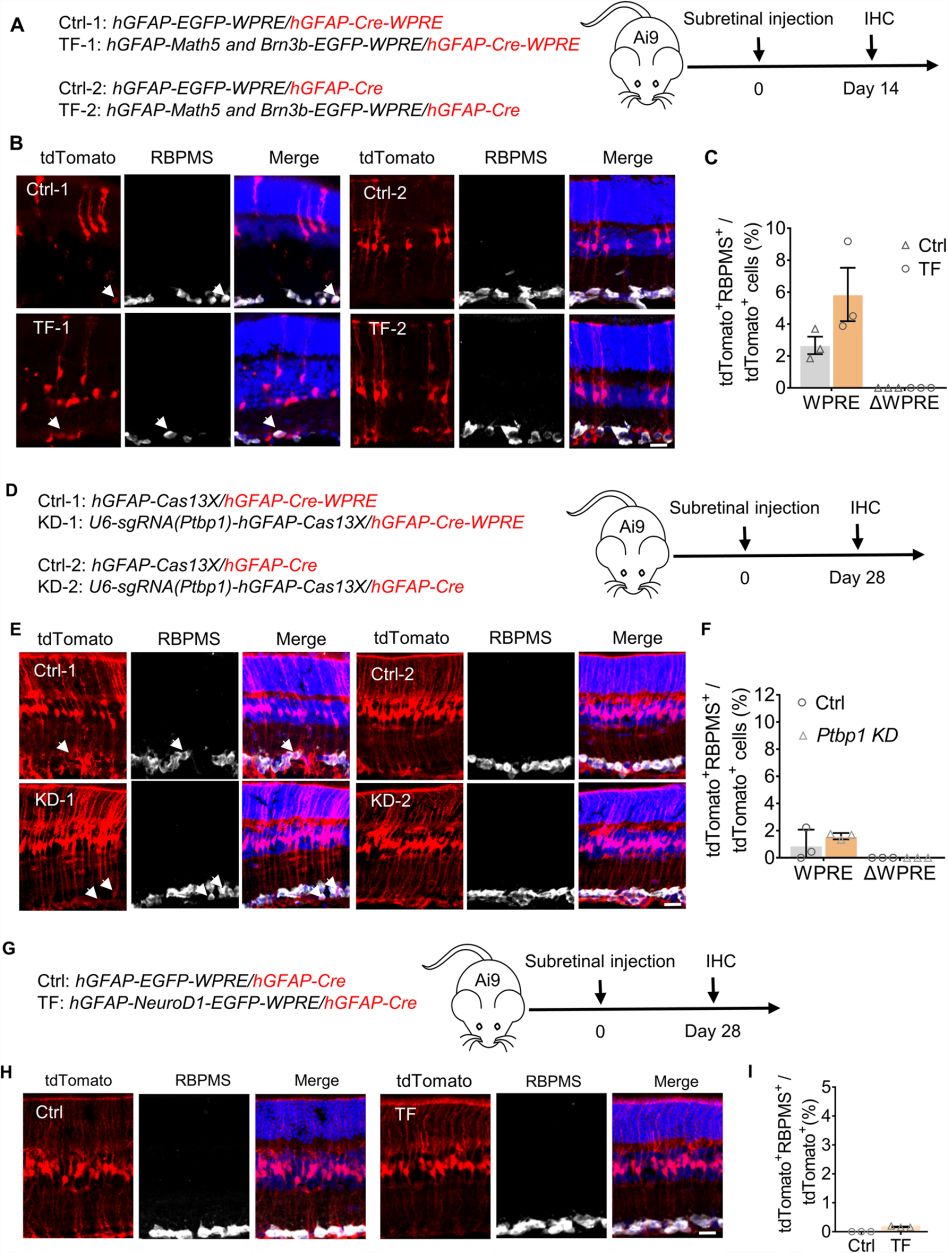
The *ΔWPRE* system fails to trace reprogrammed RGC mediated by transcription factors overexpression or knockdown. (**A**) Schematic diagram of vectors for *Math5* and *Brn3b* overexpression and injection process. (**B**) Representative images of tdTomato co-labeling with Rbpms at 2 weeks after injection. (**C**) The ratio of tdTomato-labeled RGCs among total tdTomato^+^ cells. (**D**) Schematic diagram of *Ptbp1* knockdown and injection process. (**E**) Representative images of tdTomato co-labeling with Rbpms. (**F**) The ratio of tdTomato-labeled RGCs among total tdTomato^+^ cells. (**G**) Schematic diagram of *NeuroD1* overexpression. (**H**) Representative images of tdTomato co-labeling with Rbpms. (**I**) The ratio of tdTomato-labeled RGCs among total tdTomato^+^ cells. n = 3 for each group. Arrows show tdTomato^+^ Rbpms^+^ cells in panels B, E, H. Scale Bar, 50 μm.

It has been reported that ectopic expression of several factors may change the specificity of GFAP promoter^12^. To test whether our *AAV9-hGFAP-Cre-ΔWPRE* label system was still stringent, we applied it to MG conversion with several factors. MG to RGC conversion was observed under the overexpression of Math5 and Brn3b, traced by a hGFAP-reporter-WPRE label system^8^. We labeled this Math-Brn3b-induced conversion with both our Δ*WPRE* system and the *hGFAP-Cre-WPRE* system (Fig. 3A). Using the same tracking system in the previous study^8^, the expression of *Math5* and *Brn3b* represented by the EGFP was observed in the inner nuclear layer (Supplementary Fig. 3A–C). And the colocalization of EGFP and SOX9 signal (Muller cell marker) suggested the construct of AAV-*hGFAP-Math5-T2A-Brn3b-P2A-EGFP* was successfully expressed in Muller glia (Supplementary Fig. 3D). A small proportion of tdTomato^+^Rbpms^+^ cells were detected in both overexpression and control groups with the *hGFAP-Cre-WPRE* system, which implied the leakage of the *hGFAP-Cre-WPRE* system in the RGCs again. However, no tdTomato^+^ RGCs were found with the Δ*WPRE* label system at 2 weeks after injection among thousands of tdTomato^+^ cells (control: 0/1825, overexpression: 0/1606 in Δ*WPRE* groups) (Fig. 3B,C).

To further validate the specificity of the Δ*WPRE* system, we traced the conversion of MG to RGCs mediated by Ptbp1 knockdown using *hGFAP-GFP-WPRE* similar to the previous study^7^ and the Δ*WPRE* system (Fig. 3D). We found that Ptbp1 could be repressed by Cas13X in vivo^25,26^. Although *hGFAP-GFP-WPRE* still mislabeled some RGCs both in control and *Ptbp1* knockdown groups, no tdTomato^+^ RGCs were detected using the Δ*WPRE* system (Fig. 3E,F)*.* *In* addition, ganglion axon fibers were labeled in both control and knockdown groups of the label system including the WPRE element, while no tdTomato signals were found in the Δ*WPRE* system (Supplementary Fig. 4A). These results suggested no MG reprogramming in Ptbp1-knockdown group. This observation by our MG labeling system is consistent with two studies using genetic lineage tracing to label MG and found no MG conversion in Ptbp1 knockdown or knockout group^10,11^.

Besides, NeuroD1 is considered as a prominent reprogramming factor in the nervous system^27,28^, but whether it enables the MG to RGCs conversion in the retina was not validated. We tracked this process with our Δ*WPRE* system and labeled NeuroD1 expression by EGFP (Supplementary Fig. 4B–C), and observed no tdTomato^+^ RGCs were labeled (Fig. 3G–I).

## Discussion

In this study, we developed a relatively straightforward and accessible mouse MG labeling system as AAV9-*hGFAP-Cre-*Δ*WPRE* applied in Ai9 transgenic mice. This system can label MG efficiently and specifically without obvious leakage in RGCs.

Considering the heterogeneity of MGs including many subtypes^29,30^, conclusions regarding the ability of MG to differentiate into other neuronal types require validation by labeling and detection of all MG subtypes. However, the labeling efficiency of MG by AAV-based reporters primarily depends on its transduction efficiency which can be affected by AAV tropisms, cis-element such as *WPRE*, and administration routes^16,23^. We compared the transduction patterns among different AAV serotypes using an *hGFAP-Cre-WPRE* vector and found that AAV8 and AAV9 had higher transduction efficacy for MG than AAV1, AAV2, and AAV5 (Fig. 1B–D). Other recent work showed variable specificity by different serotypes for labeling astrocytes in the brain^31^, which were in line with our results in the retina.

In addition, high specificity for tracing MG is indispensable for the exclusion of false-positive results in the investigation of MG conversion to neurons. Until now, the efficiency and specificity of AAV-based labeling of astrocytes leads to controversial results in the study of neurogenesis and reprogramming, especially in the brain^31,32^. We found that all serotypes exhibited varying degrees of leakage to RGCs in the retina (Fig. 1F). We further improved the vector by removing the *WPRE* element and found that labeling efficiency was not affected, but the proportion of leakage in RGCs decreased by least 40-fold compared to that of systems that include *WPRE* (Fig. 2E,F)*,* substantially lower than that of other reported tracing systems for astrocytes in the brain^31,33^. In addition, we also labeled over 70% MG cells at the dose of 1 × 10^10^ vg/eye on average, comparable with transgenic *Glast-CreERT2* MG tracing lineage^10,11,34^. Moreover, the system lacking *WPRE* exhibits a consistently low leakage ratio across a wide dose range, from 1 × 10^8^ to 1 × 10^10^ vg per eye, suggesting its potential reliability for tracing MG.

Using the AAV-based *hGFAP-Cre-*Δ*WPRE* system, we re-examined MG programming mediated by transcription factors reported by previous works^7,8^ and detected false-positive results. We found suppression of Ptbp1 could not induce conversion of MG to RGC with the Δ*WPRE* label system (Fig. 3D,E). The “converted RGCs” in Ptbp1-knockdown group in the previous research might be resident RGCs when using a leaky MG labeling system^35,36^. Using genetic lineage tracing system (e.g. Glast-CreERT;Rosa-CAG-LSL-Sun1-GFP), two other groups observed similar results in Ptbp1 knockdown retina as we do^10,11^, indicating our AAV-based MG tracing system showed similar specificity to genetic lineage tracing mouse models. In addition, we observed no MG conversion in Atoh7-Brn3b overexpression group using AAV-based MG tracing system, which is different from the previous work using a WPRE-containing MG-tracing system^8^. Thus, the MG conversion by Atoh7-Brn3b overexpression should be revisited using a more cautious approach in the future. It is also interesting to test *AAV9-hGFAP-Cre-*Δ*WPRE* system in animals having no genetic strains.

In summary, the *AAV9-hGFAP-Cre-*Δ*WPRE* system showed high efficiency and specificity for MG labeling, providing a useful tool for in vivo MG-lineage tracing in eye development, as well as for the development of MG-targeted therapeutics.

## Materials and methods

### Animals

All mice were housed under a 12-h light/dark cycle with water and food provided ad libitum at the Animal Center of the Institute of Neuroscience, Chinese Academy of Sciences, Shanghai, China. All animal procedures were approved by the Animal Care and Use Committee of the Institute of Neuroscience, Chinese Academy of Sciences, and all experiments were performed in compliance with relevant guidelines and recommendations, including the guide for the care and use of laboratory animals. All animal studies reported also followed the recommendations in the ARRIVE guidelines. All reporters were based on tdTomato expression in *Ai9* transgenic mice (*CAG-Loxp-stop-Loxp-tdTomato*, JAX #007,909) purchased from the Jackson Laboratory.

### AAVs and vectors

All AAV vectors were constructed by PCR-based subcloning. The 681-bp human *GFAP* promoter was used in this study, which was derived from the 2.2-kb gfa2 promoter^37^. *hGFAP-Cre-WPRE* was cloned on basis of the backbone of an AAV vector^7,38^. The vector *hGFAP-Cre-ΔWPRE* was constructed from *hGFAP-Cre-WPRE* where the *WPRE* element was removed. DNAs encoding *EGFP*, *Math5*/*Brn3b,* and *NeuroD1* were used to replace *Cre* in the backbone of *hGFAP-Cre-WPRE* to build *hGFAP-EGFP-WPRE*, *hGFAP-Math5-Brn3b-EGFP-WPRE, and hGFAP-NeuroD1-EGFP-WPRE*. Different serotypes of AAVs were packaged and titered by Gene Editing Core Facility in the Institute of Neuroscience or PackGene Company.

### Subretinal injection

Given the low labeled efficiency by the *hGFAP-Cre* AAV vector via intravitreal injections^19^, AAVs were delivered to the eyes via subretinal injection, as previously described^39,40^. For subretinal injection, adult mice were anesthetized with a mixture of zoletil and xylazine (dose: 60 μg zoletil and 10 μg xylazine per gram body weight; zoletil, Virbac; xylazine, Huamu Animal Health Products Co, Ltd. of Jilin Province, China), and pupils were dilated with tropicamide phenylephrine eye drops (Dirui, China)before injection. A small scleral incision was made using a 30G needle under a microscope (Olympus, Tokyo, Japan). Then a 32G needle on a Hamilton syringe was inserted into the subretinal space through the scleral incision. 1 μl of AAV was slowly (i.e. up to 20 s) injected into the subretinal space. After the AAV injection, the Hamilton syringe was removed and a drop of ofloxacin eye ointment was applied to cover the eye.

To test the labeling specification of different AAV serotypes, 1 μl of AAV including 1 × 10^9^ or 1 × 10^8^ vg was separately injected. To test the labeling specifications of the *hGFAP-Cre* AAV vector, 1 μl of AAV including 1 × 10^10^, 1 × 10^9^, or 1 × 10^8^ vg was separately injected. In gene transfer of transcription factors experiments, 1 μl of AAV included 5 × 10^8^ vg *hGFAP-Cre* and 1 × 10^10^ vg *hGFAP-EGFP-WPRE* in the control group, 5 × 10^8^ vg *hGFAP-Cre* and 1 × 10^10^ vg *hGFAP-Math5-Brn3b-EGFP-WPRE*, or 1 × 10^10^ vg *hGFAP-NeuroD1-EGFP-WPRE,* or *U6-sgRNA(Ptbp1)-hGFAP-Cas13X* in the overexpression or knockdown groups (Table 1). All mice were injected at 4 ~ 8 weeks old.

**Table 1:** Details of subretinal injection.

Label system	Groups	Vectors	Dose	Volume
WPRE	Control	*AAV9-hGFAP-EGFP-WPRE*	1 × 10^10^ vg	1 μl
		*AAV9-hGFAP-Cre-WPRE*	5 × 10^8^ vg	
	Math5 and Brn3b overexpression	*AAV9-hGFAP-Math5-Brn3b-EGFP-WPRE*	1 × 10^10^ vg	1 μl
		*AAV9-hGFAP-Cre-WPRE*	5 × 10^8^ vg	
	Control	*AAV9 -hGFAP-Cas13X*	1 × 10^10^ vg	1 μl
		*AAV9-hGFAP-Cre-WPRE*	5 × 10^8^ vg	
	Ptbp1 knockdown	*AAV9-U6-sgRNA(Ptbp1)-hGFAP-Cas13X*	1 × 10^10^ vg	1 μl
		*AAV9-hGFAP-Cre-WPRE*	5 × 10^8^ vg	
ΔWPRE	Control	*AAV9-hGFAP-EGFP-WPRE*	1 × 10^10^ vg	1 μl
		*AAV9-hGFAP-Cre*	5 × 10^8^ vg	
	Math5 and Brn3b overexpression	*AAV9-hGFAP-Math5-Brn3b-EGFP-WPRE*	1 × 10^10^ vg	1 μl
		*AAV9-hGFAP-Cre*	5 × 10^8^ vg	
	NeuroD1 overexpression	*AAV9- hGFAP-NeuroD1-EGFP-WPRE*	1 × 10^10^ vg	1 μl
		*AAV9-hGFAP-Cre*	5 × 10^8^ vg	
	Control	*AAV9 -hGFAP-Cas13X*	1 × 10^10^ vg	1 μl
		*AAV9-hGFAP-Cre*	5 × 10^8^ vg	
	Ptbp1 knockdown	*AAV9-U6-sgRNA(Ptbp1)-hGFAP-Cas13X*	1 × 10^10^ vg	1 μl
		*AAV9-hGFAP-Cre*	5 × 10^8^ vg	

### Immunofluorescent staining and imaging

Mouse eyes were collected after perfusing animals with 4% paraformaldehyde (PFA, Sigma) in PBS, and post-fixed for half an hour in PFA at room temperature after removing the cornea. Then, retinas were dissected and dehydrated overnight using 30% (*w/v*) sucrose in PBS and embedded in O.C.T (Sakura). Retinas with blood or bubbles in the injection site were removed.

The whole retina was sectioned into 20 μm slices by a cryostat (HM525, Thermo). All sections for each eye were collected and serially pasted into 6 microscopic slides to make sure retina planes in each slide were comparable. Sections for different cell markers staining were chosen from serial microscopic slides in the same treatment. Before staining, sections were dried for 30 min at 65℃ and then were washed in PBS 3 times for 5 min per wash. Blocking was performed in 150 μl blocking buffer (10% goat serum, and 0.1% Tween-20 in 0.1 M PBS; goat serum, Invitrogen; Tween-20, Sangon Biotech). Primary antibodies were incubated at 4℃ overnight. Details of primary antibodies used in this study were as follows: rabbit anti-RBPMS (1:200, Proteintech, 15187-1-AP); rabbit anti-SOX9 (1:200, millipore, AB5535). Sections were washed 3 × for 10 min per wash on the second day. Secondary antibodies were also incubated at 4℃ overnight. These secondary antibodies included: Alexa Fluor 647-AffiniPure goat anti-rabbit IgG (H + L) (1:500, Jackson ImmunoResearch, 111-605-003). Sections were washed 3 × for 10 min per wash on the third day. After nucleic DNA staining by 4′,6-diamidino-2-phenylindole (DAPI, sigma, D8417), sections were mounted with fluorescent anti-fade mounting medium (Southern Biotech, 0100-01). All sections were previewed by confocal microscopy (Olympus, FV3000) and those near the injection site were captured.

To co-localization of GFP and SOX9 in Figure Supplementary Fig. 3D, we first captured EGFP and tdTomato signals and marked the capture site before SOX9 staining. Then we stained SOX9 signals and captured it in the same position.

### Cell counting

All retina sections in each microscopy slide were previewed by confocal microscopy and the plane near the injection site was chosen to capture images. Images were processed using Image J^7^. Cells were counted in the software of Image J. Transduction area ratio was assessed by: Transduction area = (transduction length including labeled cells* inner nuclear layer (INL) thickness)/(total retina length* INL thickness). To accurately test the labeling specification of the *hGFAP-Cre* AAV vector, 3 eye sections near the injection site for each retina were chosen to count cells, and their average values were used for analysis. In other treatments, 1 eye section near the injection site were chosen to count cells.

### Statistical analysis

For the comparison of the two groups, independent Student’s *t* *tests* were performed by Welch’s test. Multiple *t* *tests* were used for multiple comparisons and statistical significance was determined using the Holm-Sidak method. P values < 0.05 were considered statistically significant. Values were presented as mean ± standard error mean (SEM).

## Supplementary Information

Supplementary Information.

Supplementary Figures.

## Data Availability

All data generated or analyzed during this study are included in this published article and its supplementary information file.
