# Supplementary material for: Develop an efficient and specific AAV-based labeling system for Muller glia in mice
Source: Sci Rep. 2022 Dec 27;12:22410. doi: 10.1038/s41598-022-27013-0 (PMC9794687; doi:10.1038/s41598-022-27013-0)

Develop an efficient and specific AAV-based labeling system for Muller glia in mice

Yanxia Gao[1,#], Kailun Fang[1,#], Zixiang Yan[2,#], Haiwei Zhang[1,#], Guannan Geng[1], Weiwei Wu[3], Ding Xu[4], Heng Zhang[5], Na Zhong[1], Qifang Wang[1], Minqing Cai[1], Erwei Zuo[2], Hui Yang[1,*]

[1]Institute of Neuroscience, CAS Center for Excellence in Brain Science and Intelligence Technology, Shanghai Research Center for Brain Science and Brain-Inspired Intelligence, Chinese Academy of Sciences, Shanghai, China.

[2]Shenzhen Branch, Guangdong Laboratory for Lingnan Modern Agriculture, Genome Analysis Laboratory of the Ministry of Agriculture, Agricultural Genomics Institute at Shenzhen, Chinese Academy of Agricultural Sciences, Shenzhen, China.

[3]Huigene Therapeutics Inc., Shanghai, China.

[4]Department of Vascular Surgery, Changhai Hospital, Navy Medical University, Shanghai, P.R. China.

[5]Department of Vascular Surgery, The Affiliated Hospital of Qingdao University, Qingdao, China.

[#]These authors contributed equally.

[*]Correspondence should be addressed to **H.Y.** (huiyang@ion.ac.cn)

Dr. Hui Yang, Senior Investigator, Shanghai Research Center for Brain Science and Brain-Inspired Intelligence, Chinese Academy of Sciences.

Address: 320 Yue Yang Road Shanghai, 200031 P.R.China.

Figure legends

Supplementary figure 1 AAV-based systems co-label a small proportion of RGCs along with MG, related to figure 1

(A) Representative images of transduction with different AAV serotypes at the dose of $1x10^8$ vg/eye. Scale Bar, 200 μm.

(B) The average number of tdTomato-labeled cells in each eye section.

(C) Representative images of co-localization of tdTomato signal with RBPMS in the retina. Arrows show tdTomato $^+$RBPMS$^+$ cells. Scale Bar, 50 μm.

Supplementary Figure 2 Reduced leakage in the RGCs with the ΔWPRE system, related to figure 2

(A) Representative images of tdTomato co-labeling with the SOX9 MG marker in different densities of labeled MG in retina sections.

(B) The ratio of tdTomato-labeled MG among total labeled cells (left) and MG (right). n = 6~7 retina for each group.

(C) Venn diagram of the average number tdTomato-labeled cells and MG in each section.

(D) Representative images of transduction with an alternative AAV9 batch at the dose of $1x10^9$ vg/eye. Scale Bar, 200 μm.

(E) The average number of tdTomato-labeled cells in each eye section.

(F) Representative images of co-localization of tdTomato signal and RBPMS with different batches of AAVs. Arrows show tdTomato $^+$RBPMS$^+$ cells.

(G) The ratio of tdTomato-labeled MG among total labeled cells.

n = 3 retina for each group in panel E and G. Scale bar is 50 μm in panel A and F.

Supplementary figure 3 The ΔWPRE system fails to trace reprogrammed RGC by *Math5* and *Brn3b* overexpression, related to figure 3

(A) Schematic diagram of vectors for *Math5* and *Brn3b* overexpression and injection process.

(B) Co-localization of transcription factors (labeled by EGFP) and the tdTomato-labeled system.

(C) The ratio of tdTomato$^+$EGFP$^+$ cells among total tdTomato$^+$ cells. n = 3 for each group.

(D) Co-localization of GFP and SOX9 signals in *Math5* and *Brn3b* overexpression group.

Scale bar is 50 μm in panel B and D.

Supplementary figure 4 The ΔWPRE system fails to trace reprogrammed RGC by *Ptbp1* knockdown or *NeuroD1* overexpression, related to figure 3

(A) The tdTomato signal in the ganglion axon fibers after *Ptbp1* knockdown. Dotted lines show the contour of the ganglion axon fiber. Scale bar, 500 μm.

(B) Co-localization of NeuroD1 (labeled by EGFP), the tdTomato-labeled system and RBPMS following *NeuroD1* overexpression. Scale bar, 50 μm.

(C) The ratio of tdTomato$^+$EGFP$^+$ cells among total tdTomato$^+$ cells. n = 3 for each group.

Supplementary figure 1 AAV-based systems co-label a small proportion of RGCs along with MG, related to figure 1

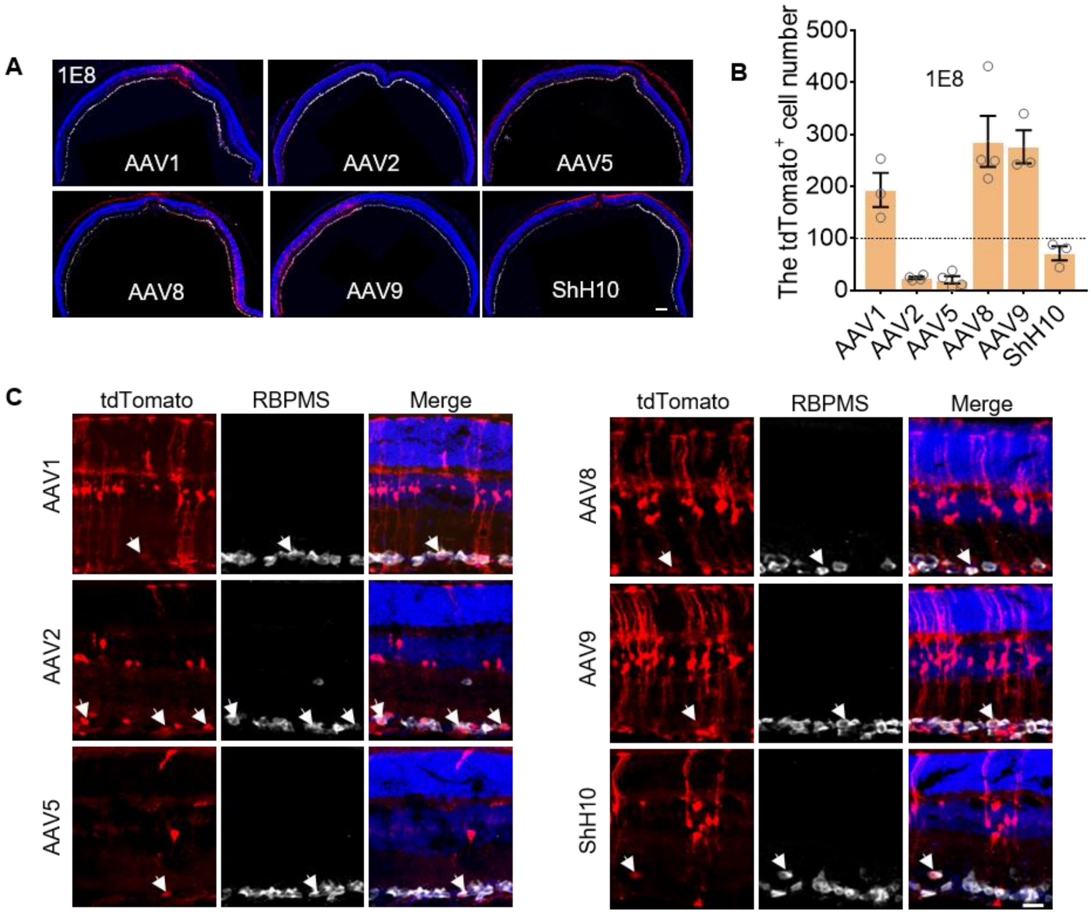

Supplementary Figure 2 Reduced leakage in the RGCs with the ΔWPRE system, related to figure 2

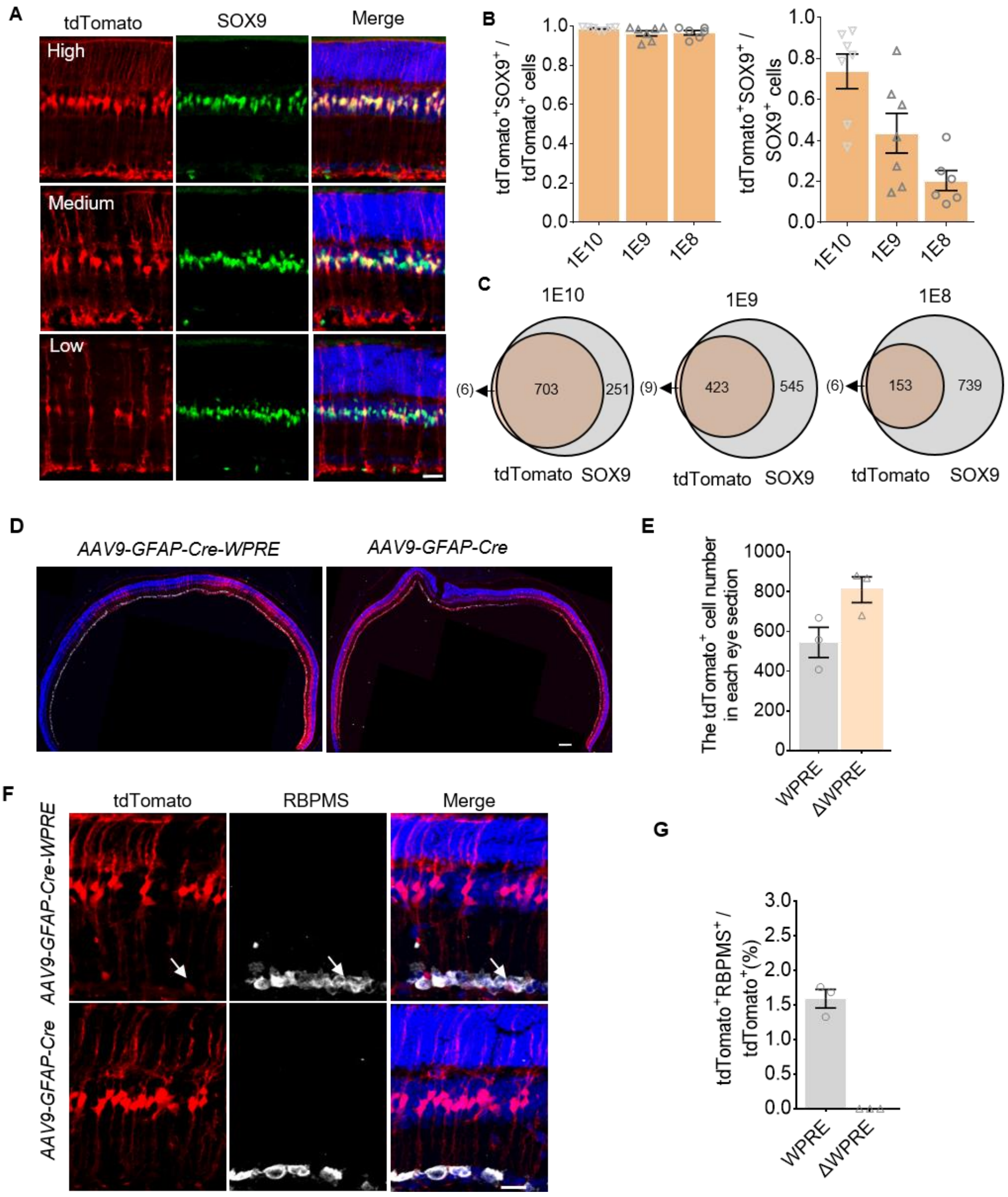

Supplementary figure 3 The ΔWPRE system fails to trace reprogrammed RGC by *Math5* and *Brn3b* overexpression, related to figure 3

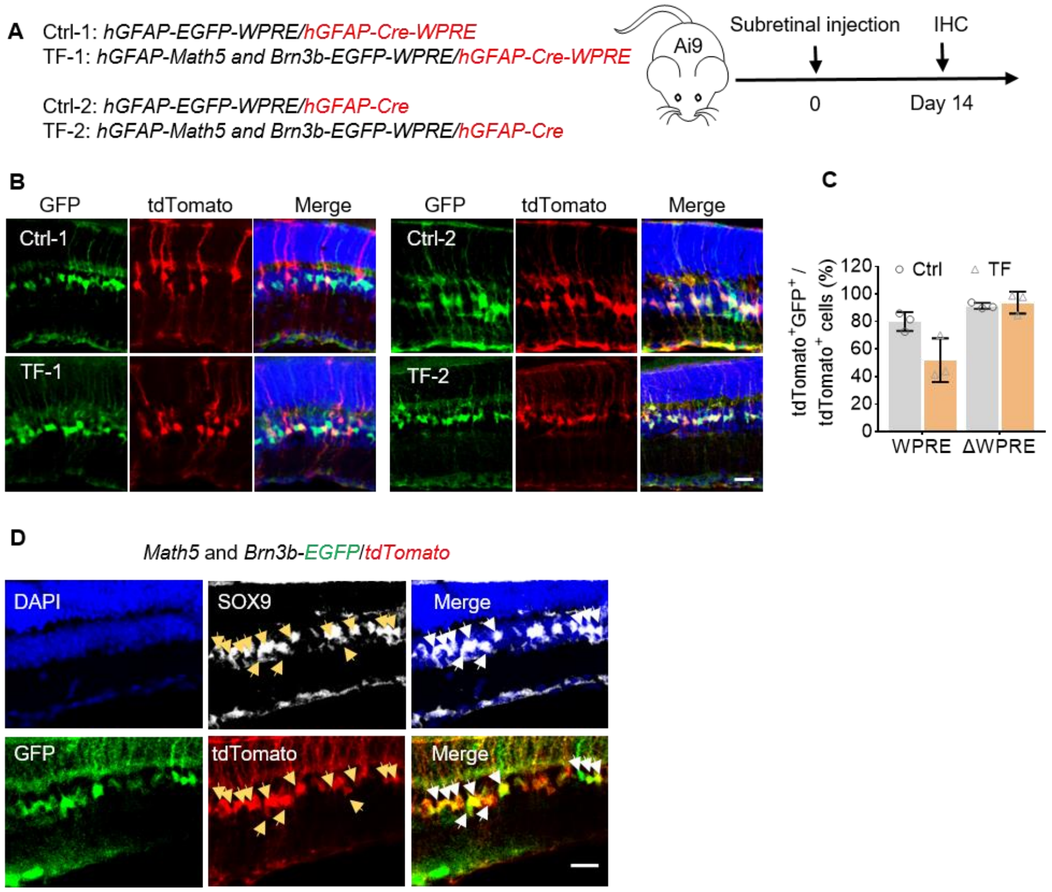

Supplementary figure 4 The ΔWPRE system fails to trace reprogrammed RGC by *Ptbp1* knockdown or *NeuroD1* overexpression, related to figure 3

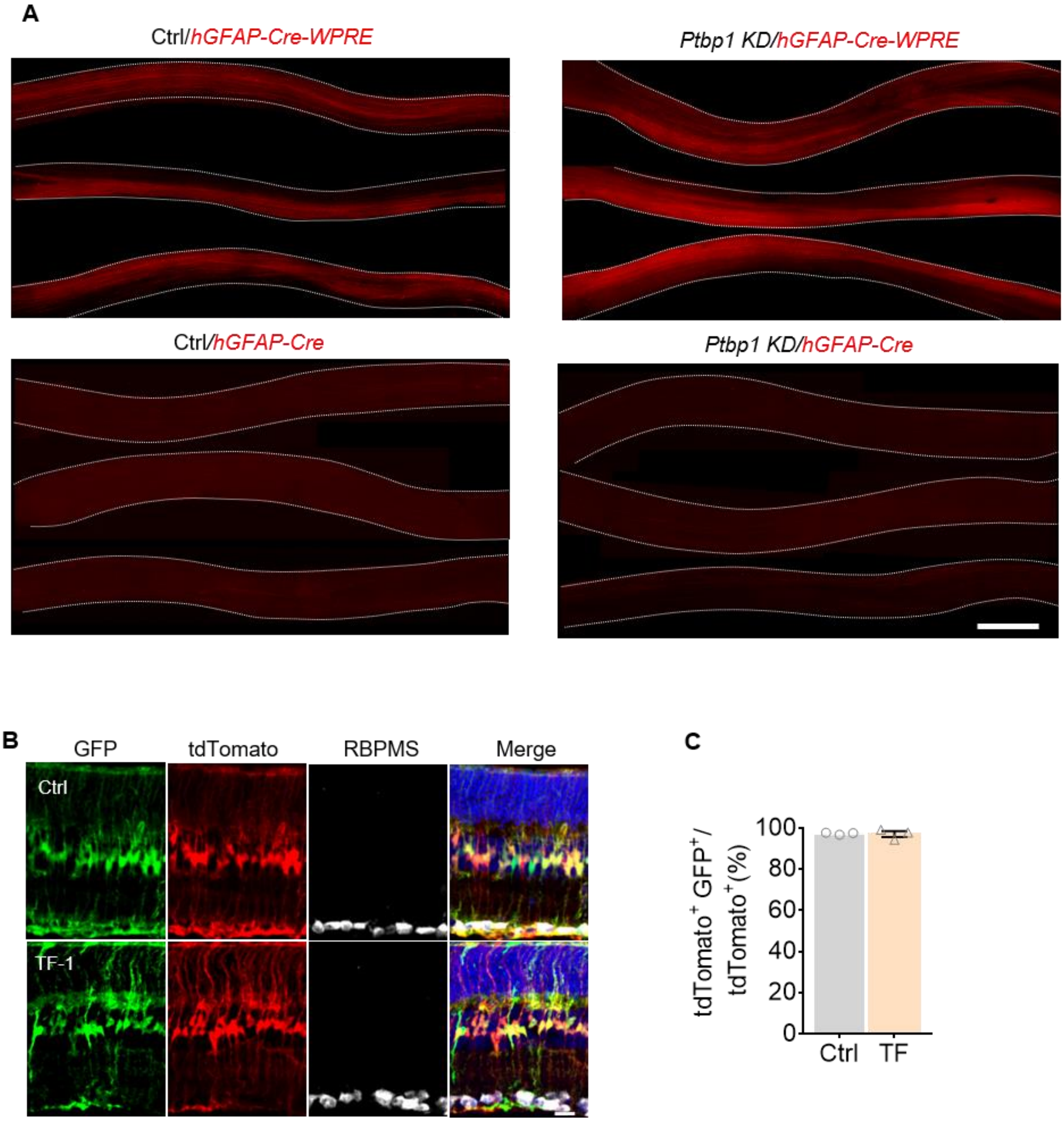

Supplement: Supplementary file 2 — Supplementary Figures. [file 41598_2022_27013_MOESM2_ESM.pdf]
